# Supplementary figures and images for: Exploring the trends of adaptation and evolution of sclerites with regards to habitat depth in sea pens
Source: PeerJ. 2022 Sep 21;10:e13929. doi: 10.7717/peerj.13929 (PMC9508890; doi:10.7717/peerj.13929)

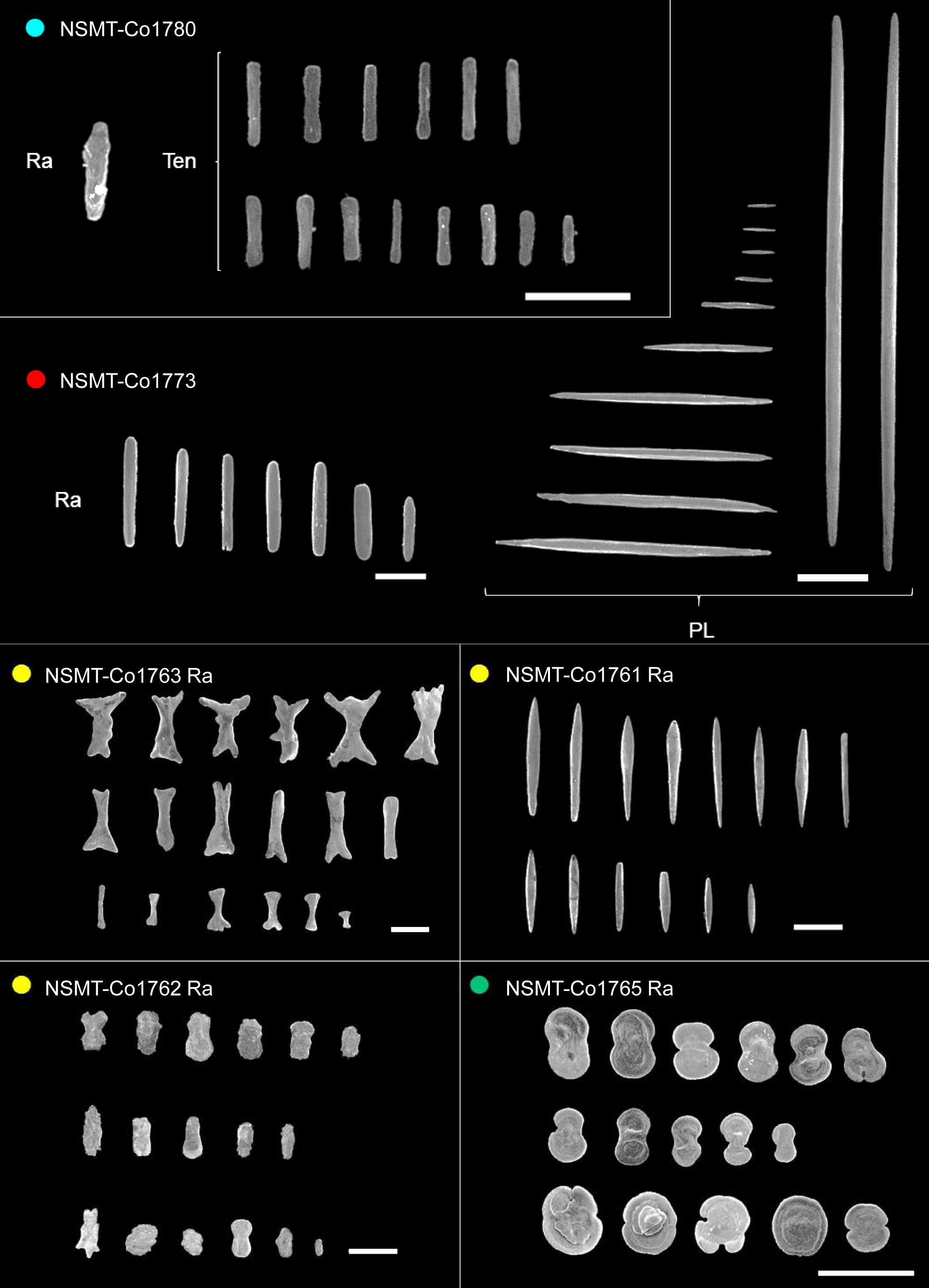

Supplement: Supplemental Information 6 — Each circle color indicates each family; light blue: Virgulariidae, red: Pennatulidae, yellow: Veretillidae, green: Kophobelemnidae. Numbers are specimen numbers. Abbreviations; ten, tentacles; Ra, Rachis. All scale bars = 100 µm. [file peerj-10-13929-s006.png]

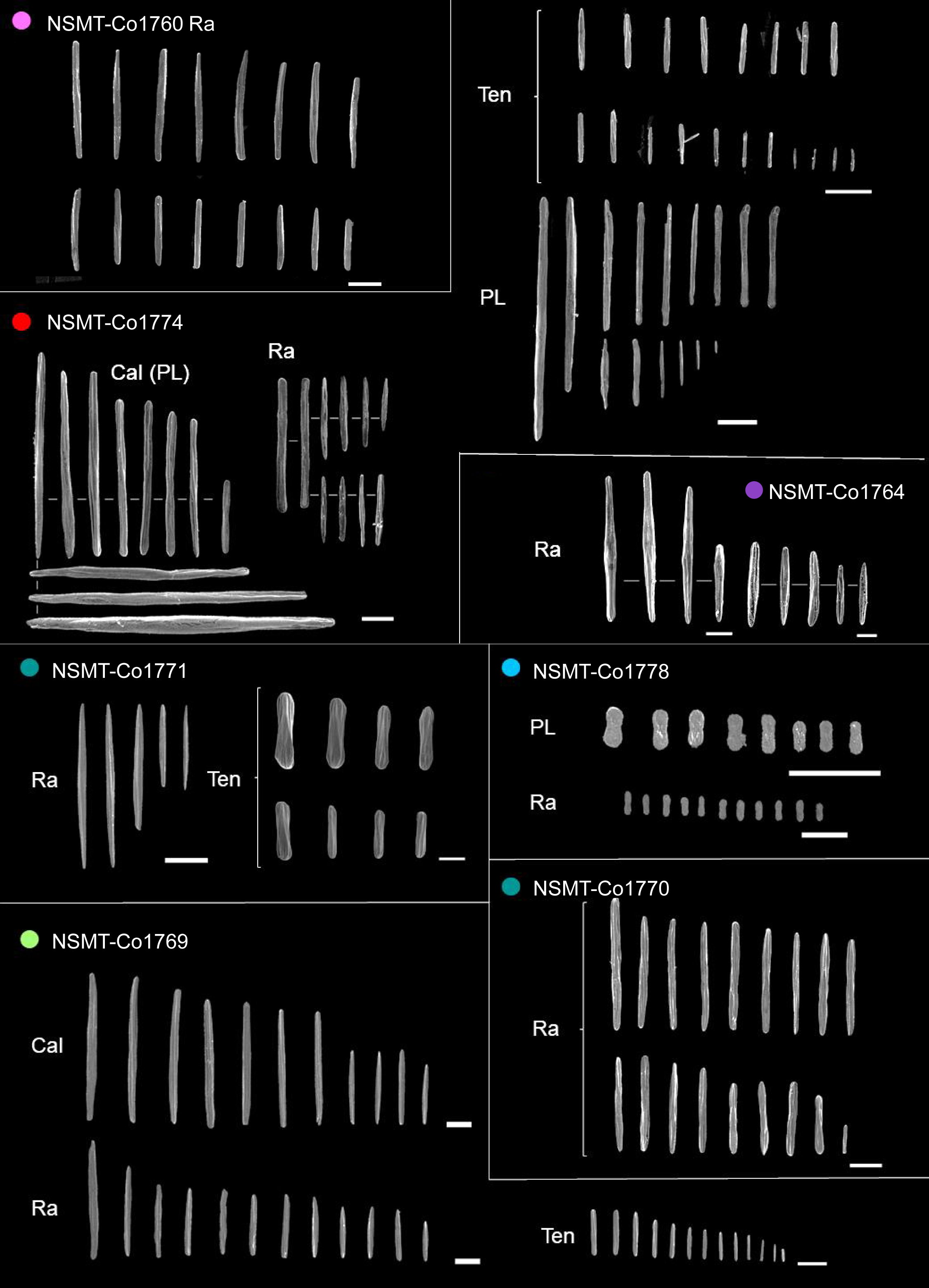

Supplement: Supplemental Information 7 — Each circle color indicates each family; yellow green: Stachyptilidae, pink: Renillidae, red: Pennatulidae, light blue: Virgulariidae, blue green: Protoptilidae, purple: Echinoptilidae. Numbers are specimen numbers. Abbreviations; cal, calyces; PL, polyp leaf; ten, tentacles; Ra, rachis. Scale bars = 50 µm (NSMT-Co1764 Ra including calyx of siphonozooids (Right)), 100 µm (NSMT-Co1769 Cal, Ra, Ten; NSMT-Co1774 Ten, Cal, Ra; NSMT-Co1760 Ra; NSMT-Co1778 PL, Ra; NSMT-Co1764 Ra including calyx (Left)), 200 µm (NSMT-Co 1774 PL; NSMT-Co 1771 Ra). [file peerj-10-13929-s007.png]

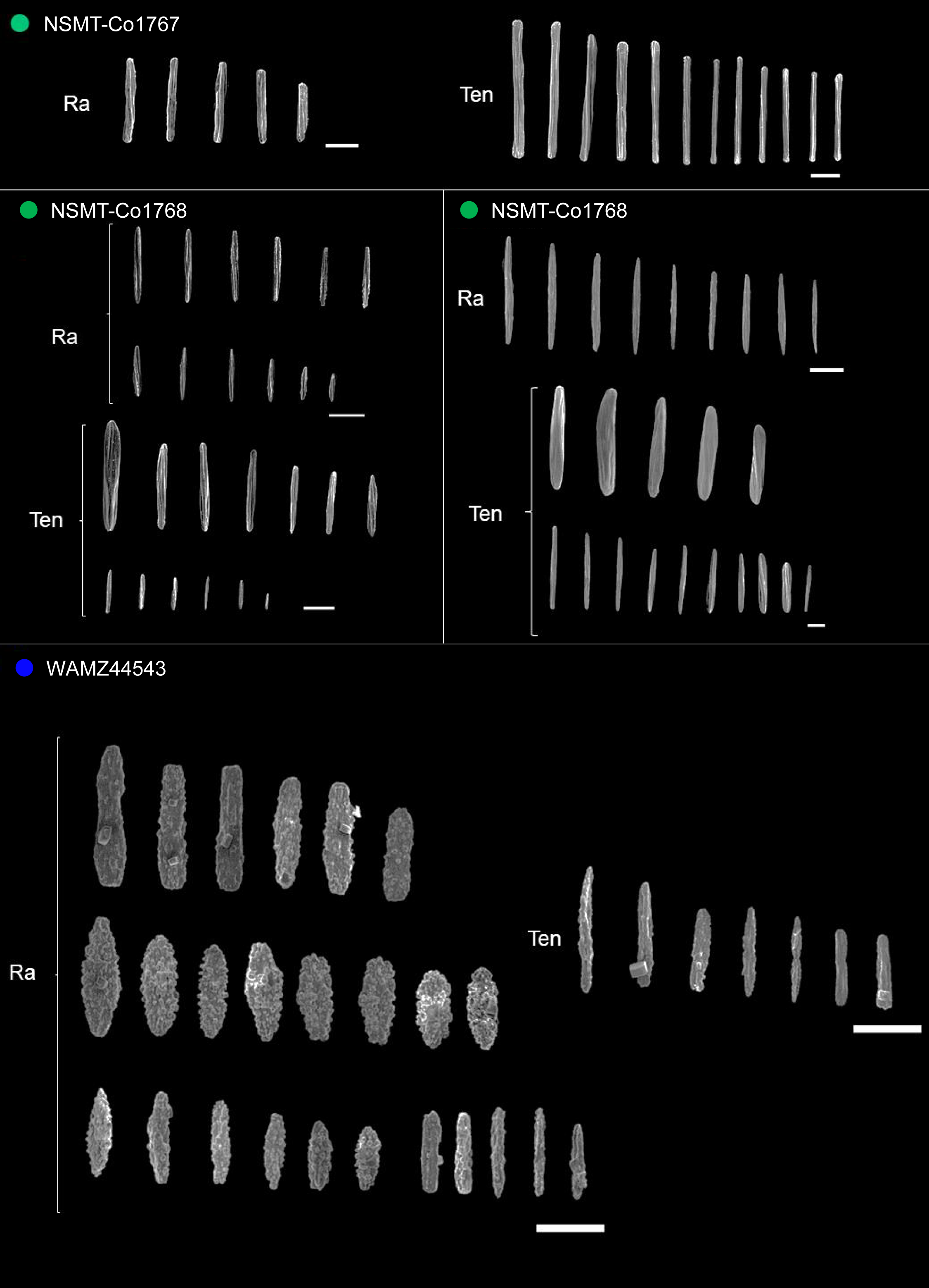

Supplement: Supplemental Information 8 — Each circle color indicates each family; green: Kophobelemnidae; blue: Pennatulacea sp. Numbers are specimen numbers. Abbreviations; ten= tentacles, Ra= Rachis. All scale bars = 100 µm. [file peerj-10-13929-s008.png]
